# Six Novel O Genotypes from Shiga Toxin-Producing *Escherichia coli*

**DOI:** 10.3389/fmicb.2016.00765

**Published:** 2016-05-20

**Authors:** Atsushi Iguchi, Sunao Iyoda, Kazuko Seto, Hironobu Nishii, Makoto Ohnishi, Hirohisa Mekata, Yoshitoshi Ogura, Tetsuya Hayashi

**Affiliations:** ^1^Department of Animal and Grassland Sciences, Faculty of Agriculture, University of MiyazakiMiyazaki, Japan; ^2^Department of Bacteriology I, National Institute of Infectious DiseasesTokyo, Japan; ^3^Division of Bacteriology, Osaka Prefectural Institute of Public HealthOsaka, Japan; ^4^Organization for Promotion of Tenure Track, University of MiyazakiMiyazaki, Japan; ^5^Center for Animal Disease Control, University of MiyazakiMiyazaki, Japan; ^6^Department of Bacteriology, Faculty of Medical Sciences, Kyushu UniversityFukuoka, Japan

**Keywords:** *E. coli*, O serogroup, genotyping techniques, PCR, STEC

## Abstract

Serotyping is one of the typing techniques used to classify strains within the same species. O-serogroup diversification shows a strong association with the genetic diversity of O-antigen biosynthesis genes. In a previous study, based on the O-antigen biosynthesis gene cluster (O-AGC) sequences of 184 known *Escherichia coli* O serogroups (from O1 to O187), we developed a comprehensive and practical molecular O serogrouping (O genotyping) platform using a polymerase chain reaction (PCR) method, named *E. coli* O-genotyping PCR. Although, the validation assay using the PCR system showed that most of the tested strains were successfully classified into one of the O genotypes, it was impossible to classify 6.1% (35/575) of the strains, suggesting the presence of novel O genotypes. In this study, we conducted sequence analysis of O-AGCs from O-genotype untypeable Shiga toxin-producing *E. coli* (STEC) strains and identified six novel O genotypes; OgN1, OgN8, OgN9, OgN10, OgN12 and OgN31, with unique *wzx* and/or *wzy* O-antigen processing gene sequences. Additionally, to identify these novel O-genotypes, we designed specific PCR primers. A screen of O genotypes using O-genotype untypeable strains showed 13 STEC strains were classified into five novel O genotypes. The O genotyping at the molecular level of the O-AGC would aid in the characterization of *E. coli* isolates and will assist future studies in STEC epidemiology and phylogeny.

## Introduction

Serotyping is a standard method for subtyping of *Escherichia coli* strains in taxonomical and epidemiological studies (Orskov and Orskov, 1984). In particular, the identification of strains of the same O serogroup is essential in outbreak investigations and surveillance for identifying the diffusion of a pathogenic clone (Frank et al., 2011; Luna-Gierke et al., 2014; Terajima et al., 2014; Heiman et al., 2015). Thus far, the World Health Organization Collaborating Centre for Reference and Research on *Escherichia* and *Klebsiella*, which is based at the Statens Serum Institut (SSI) in Denmark^1^, has recognized 185 *E. coli* O serogroups. These are designated O1 to O188 (publication of O182 to O188 is pending) and include three pairs of subgroups, O18ab/ac, O28ab/ac, and O112ab/ac; and six missing numbers, O31, O47, O67, O72, O93, and O122 (Orskov and Orskov, 1992; Scheutz et al., 2004).

O-serogroup diversification shows a strong association with the genetic diversity of O-antigen biosynthesis genes. In *E. coli*, the genes required for O-antigen biosynthesis are clustered at a chromosomal locus flanked by the colanic acid biosynthesis gene cluster (*wca* genes) and the histidine biosynthesis (*his*) operon. Sequence comparisons of O-antigen biosynthesis gene clusters (O-AGCs) indicate a variety of genetic structures (DebRoy et al., 2011a). In particular, sequences from O-antigen processing genes (*wzx*/*wzy* and *wzm*/*wzt*) located on the O-AGCs are highly variable and can be used as gene markers for the identification of O serogroups via molecular approaches. So far, several studies have reported genetic methodologies allowing rapid and low-cost O-typing of isolates (Coimbra et al., 2000; Beutin et al., 2009; Bugarel et al., 2010; Wang et al., 2010, 2014; DebRoy et al., 2011b; Fratamico and Bagi, 2012; Quiñones et al., 2012; Geue et al., 2014). In a previous study (Iguchi et al., 2015a), we analyzed the O-AGC sequences of 184 known *E. coli* O serogroups (from O1 to O187), and organized 162 DNA-based O serogroups (O-genotypes) on the basis of the *wzx*/*wzy* and *wzm*/*wzt* sequences. Subsequently we presented a comprehensive molecular O-typing scheme: an *E. coli* O-genotyping polymerase chain reaction (ECOG-PCR) system using 20 multiplex PCR sets containing 162 O-genotype-specific PCR primers (Iguchi et al., 2015b).

The Shiga toxin-producing *E. coli* (STEC) constitute one of the most important groups of food-borne pathogens, as they can cause gastroenteritis that may be complicated by hemorrhagic colitis or hemolytic-uremic syndrome (HUS; Tarr et al., 2005). O157 is a leading STEC O serogroup associated with HUS (Terajima et al., 2014; Heiman et al., 2015) and other STEC O serogroups, including O26, O103, O111, O121 and O145, are also recognized as significant food-borne pathogens worldwide (Johnson et al., 2006). Additionally, unexpected STEC O serogroups have sometimes emerged to cause sporadic cases or outbreaks. For example, STEC O104:H4 was responsible for a large food-borne disease outbreak in Europe in Buchholz et al. (2011). For such various O-serogroups, ECOG-PCR is an accurate and reliable approach for subtyping *E. coli* isolates from patients and contaminated foods (Iguchi et al., 2015b; Ombarak et al., 2016). However, as our previous studies indicated, some of the tested strains were not classified into any of the known O genotypes, suggesting the presence of novel O genotypes (Iguchi et al., 2015b).

Here, we analyzed the O-AGCs from genetically untypeable STEC strains (including strains from patients with diarrhea and hemorrhagic colitis) by the ECOG-PCR. By comparing sequences we revealed six novel O-genotypes and developed specific-PCRs for each novel O-genotype.

## Materials and Methods

### O Serogrouping/O Genotyping

O serogroup were determined by agglutination tests in microtiter plates using commercially available pooled and single antisera against all recognized *E. coli* O antigens (O1 to O187; SSI Diagnostica, 156 Hillerød, Denmark). O genotypes were determined by ECOG-PCR as described in our previous study (Iguchi et al., 2015b). *Salmonella enterica* O42 (SSI Diagnostica) and *Shigella boydii* type 13 (Denka Seiken Co. Ltd., Japan) single antisera were also used to test for the agglutination reaction.

### Source Sequences of Novel O-Genotypes

The O-AGC sequences were determined from six O-genotype untypeable (OgUT) STEC strains, of which four were serologically typeable (O1, O39, O40, and O141) and two others were untypeable (OUT) strains (**Table 1**). All strains were isolated from human feces (including patients with diarrhea and hemorrhagic colitis) in Japan from 2008 to 2012. The O-AGC sequences flanked by *wcaM* and *hisI* were extracted from draft genome sequences determined using an Illumina MiSeq sequencer (Illumina, San Diego, CA, USA), as previously described (Ogura et al., 2015). Identification and functional annotation of the coding sequences were performed based on the results of homology searches against the public non-redundant protein database using BLASTP. Six O-AGC sequences reported in this paper have been deposited in the GenBank/EMBL/DDBJ database (accession no. LC125927-LC125932).

**Table 1:** Shiga toxin-producing *E. coli* (STEC) strains used for sequencing of the O-AGC.

Strain ID	Source (case*^a^*)	Year	O serogroup	O genotype	Novel O genotype	*stx1*	*stx2*	*eae*	Reference
090823	Human (D)	2009	O1	OgUT	OgN10	+	-	-	Iguchi et al., 2015b
100998	Human (BD)	2010	O39	OgUT	OgN31	-	+	-	Iguchi et al., 2015b
121862	Human (D)	2012	O40	OgUT	OgN1	-	+	-	Iguchi et al., 2015b
102755	Human (BD)	2010	O141	OgUT	OgN8	-	+	-	Iguchi et al., 2015b
OT-11	Human (AC)	2008	OUT	OgUT	OgN9	+	-	+	In this study
EHO-67	Human (D)	2011	OUT	OgUT	OgN12	+	-	-	In this study

*^a^AC, asymptomatic carrier; D, diarrhea; BD, bloody diarrhea.*

### Sequence Comparisons

The *wzx*/*wzy* sequences from O-serogroup strains (Iguchi et al., 2015a) and OX-groups reference strains (DebRoy et al., 2016) were used. Additionally, O-AGC sequences from O116 (AB812051; Iguchi et al., 2015a), O1 (GU299791; Li et al., 2010), O39 (AB811616; Iguchi et al., 2015a), O141 (DQ868765; Han et al., 2007), O40 (EU296417; Liu et al., 2008), *S. enterica* O42 (JX975340; Liu et al., 2014), and *S. boydii* type 13 (AY369140; Feng et al., 2004) were also used. Multiple alignments of DNA and amino acid sequences were constructed by using the CLUSTAL W program (Thompson et al., 1994). Phylogenetic trees were constructed by using the neighbor-joining algorithm using MEGA5 software (Tamura et al., 2007). Homology comparisons of paired sequences were performed by using the In Silico Molecular Cloning Genomics Edition (In Silico Biology, Inc., Yokohama, Japan).

### PCR for Identifying Novel O-Genotypes

Polymerase chain reaction primers for specifically identifying novel O genotypes were designed (**Table 2**) and their specificities were evaluated by using 185 O-serogroup reference strains (O1–O188) from SSI using the following PCR conditions. Genomic DNA from *E. coli* strains was purified using the Wizard Genomic DNA purification kit (Promega, Madison, WI, USA) or DNeasy Blood & Tissue Kit (QIAGEN, Hilden, Germany) according to the manufacturer’s instructions. PCRs were performed using 10 ng/μl of template DNA. PCR was performed as follows: each 30-μl reaction mixture contained 2 μl of genomic DNA, 6 μl of 5× Kapa *Taq* buffer, dNTP mix (final concentration, 0.3 mM each), MgCl_2_ (final concentration, 2.5 mM), primers (final concentration, 0.5 μM each), and 0.8 U of Kapa *Taq* DNA polymerase (Kapa Biosystems, Woburn, MA, USA). The thermocycling conditions were: 25 cycles of 94°C for 30 s, 58°C for 30 s, and 72°C for 1 min. PCR products (2 μl) were electrophoresed in 1.5% agarose gels in 0.5× TBE (25 mM Tris borate, 0.5 mM EDTA), and photographed under UV light after the gel was stained with ethidium bromide (1 mg/ml).

**Table 2:** Polymerase chain reaction (PCR) primer sequences for identification of six novel O genotypes.

O genotype	Primer name	Target gene	Sequence (5′–3′)	Size (bp)
OgN1	OgN1_PCR_F	*wzy*	GGTTCCCTGTTGCCAATGGT	525
	OgN1_PCR_R		GAGACGAACGTGCAGAAACCA	
OgN8	OgN8_PCR_F	*wzy*	AACCTTCGCTATGATGGGGG	940
	OgN8_PCR_R		CTTTACCAGGGATGCTCCGA	
OgN9	OgN9_PCR_F	*wzy*	AAGGTTGGTAGCGTAGGGGA	423
	OgN9_PCR_R		CTCGTATTTCGCCCCCATTC	
OgN10	OgN10_PCR_F	*wzy*	TGGTGCTGTGTGCTACCATTT	892
	OgN10_PCR_R		AAAGCCAGCCTTAAATCGGA	
OgN12	OgN12_PCR_F	*wzy*	TTGTGGCACCTGATCCTGCT	223
	OgN12_PCR_R		GCACATGCTAACCCTGCTCTT	
OgN31	OgN31_PCR_F	Glycosyltransferase	GCCATAAAAAGAGCAAGGGGG	311
	OgN31_PCR_R		GGGGCAGCTGAAAACCAATC	

### Distributional Survey of Novel O-Genotypes

Thirty five O-serogrouped *E. coli* strains from our previous study (Iguchi et al., 2015b), whose O genotypes were not identified by ECOG-PCR were used for screening novel O-genotypes by the PCR method designed in this study. The prevalence of *stx1*, *stx2* (Cebula et al., 1995) and *eae* (Oswald et al., 2000) genes in the tested STEC strains was determined by the PCR.

## Results

### Novel O-Genotypes

**FIGURE 1:**
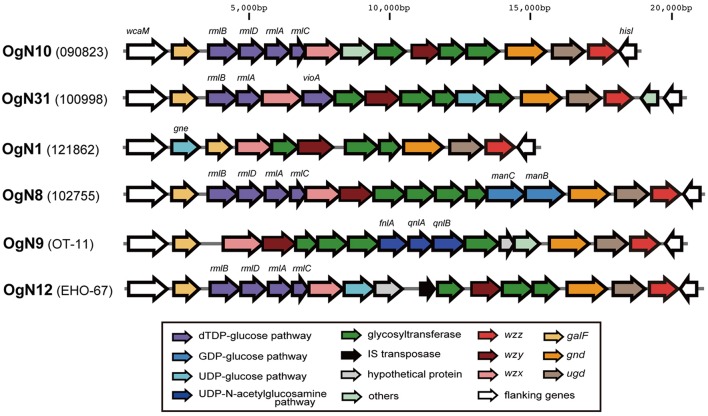
**Six novel O-AGCs from STEC strains.** The O-AGCs flanked by *wcaM* and *hisI* were extracted from draft genome sequences.

**FIGURE 2:**
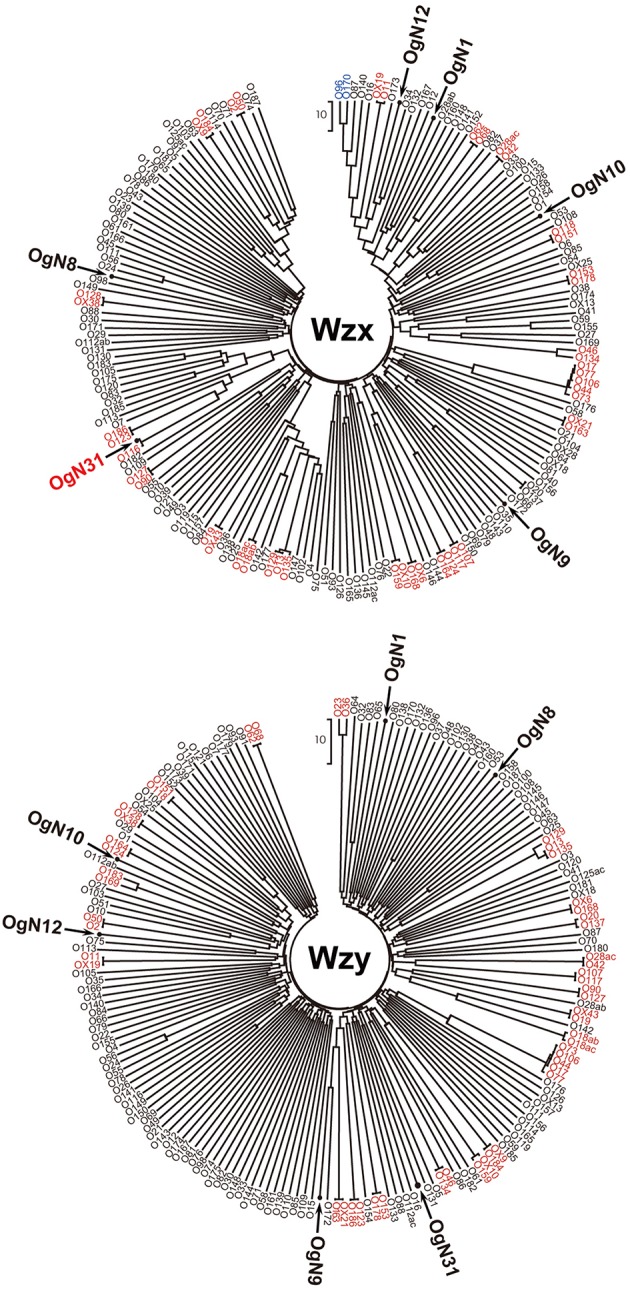
**Phylogenetic analysis of Wzx and Wzy homologs from six novel O-AGCs, *Escherichia coli* O-serogroup strains and OX-groups reference strains based on the amino acid sequences (except for *wzy* of OX25, which is not found in the O-AGC of OX25).** Pairs or groups of homologs with ≥95% DNA sequence identity are indicated in red, and ≥70% identity is indicated in blue. Other homologs with low sequence homologies (less than 70%) are indicated in black.

**FIGURE 3:**
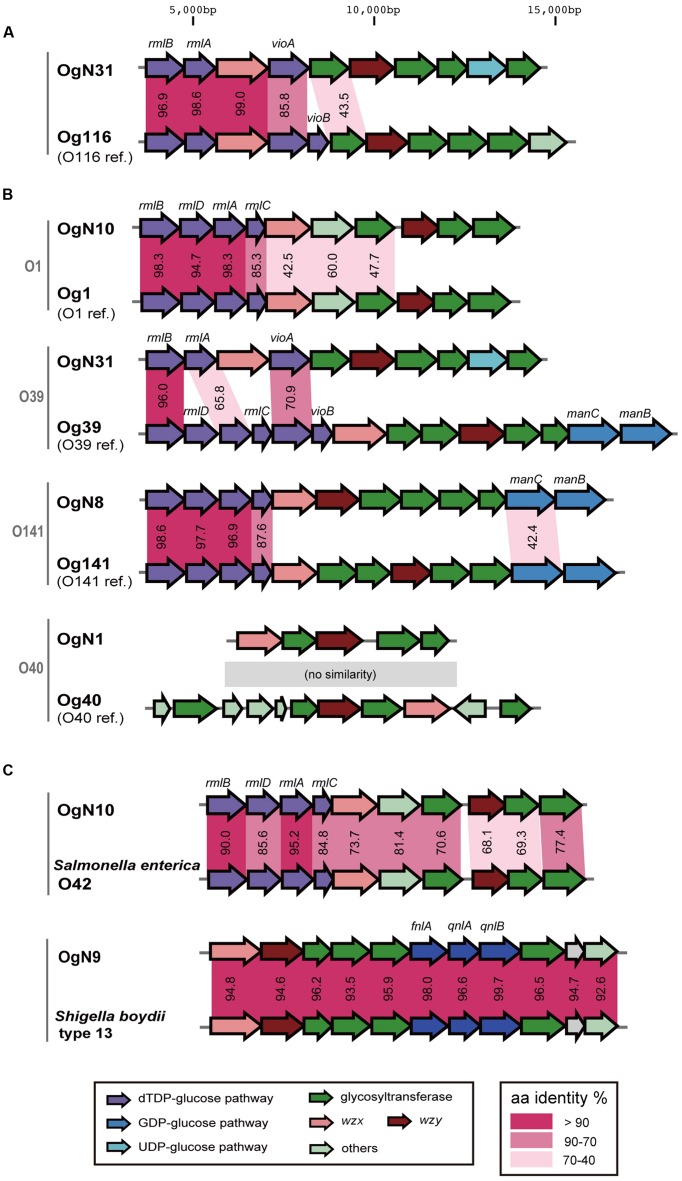
**Comparison of O-AGCs. (A)** The pair of O-AGCs, OgN31 and O116. **(B)** Four pairs that were serologically agglutinated with the same O antisera carried different types of O-AGCs. Lower genes show the O-AGCs from O-serogroup strains. **(C)** Similar O-AGCs in strains of other genera. Amino acid sequence identities (%) between homologs are shown in the middle.

Six types of O-AGC were identified from OgUT STEC strains (**Figure 1**). Four O-AGCs (named OgN10, OgN31, OgN1, and OgN8 genotypes) were obtained from strains that were serologically classified into O1, O39, O40, and O141, respectively (**Table 1**). Two others (named OgN9 and OgN12 genotypes) were obtained from strains that were both serologically and genetically unclassified into any groups (**Table 1**). Actually, the OgN9 strain did not react with any particular antiserum, and OgN12 showed identical agglutination titers with O34 and O140 antisera, which resulted in OUT classsification. OgN8, OgN10, and OgN12 carried *rmlBDAC* for the synsthesis of deoxythymidine diphosphate (dTDP)-L-rhamnose, and OgN31 carried *rmlBA*-*vioA* for dTDP viosamine synthesis (**Figure 1**). OgN9 carried *fnlA-qnlBC* for UDP-*N*-acetyl-L-quinovosamine (UDP-L-QuiNAc) synthesis (**Figure 1**). All novel O-AGCs carried the *wzx/wzy* O-antigen processing genes (**Figure 1**). The *wzx/wzy* sequences from OgN O-AGCs were compared with those from 171 O-serogroup strains and 11 OX-group reference strains, indicating that their sequences were unique compared to those from known O-AGCs (less than 70% DNA sequence identity of closest pairs), except for *wzx* of OgN31 (**Figure 2**). The sequence of OgN31_*wzx* was 98.7% identical in DNA sequence (99.0% amino acid sequence identity) to that of O116. Sequence comparison of O-AGCs revealed that the left region including *wzx* and genes for the d-TDP glucose pathway was conserved between OgN31 and Og116, and the right region including the *wzy* and glycosyltransferase genes was unique (less than 40% DNA sequence identity) in each O-AGC (**Figure 3A**). O-AGC gene sets from four pairs with members of different genotypes that agglutinated with the same O antisera were compared (**Figure 3B**). Between OgN10 and Og1 (from O1 strain), and between OgN8 and Og141 (from O141 strain), *rmlBDAC* genes were highly conserved in both O-AGCs, while other genes including *wzx* and *wzy* were diversified (less than 70% DNA sequence identity). Between OgN31 and Og39 (from O39 strain), different types of sugar biosynthesis genes were located on each O-AGC (*rmlBA*-*vioA* on OgN31, and *rmlBDAC-vioAB* and *manCB* on Og39). There was no genetic similarity between OgN1 and Og40 (from O40 strain). From these results, we were convinced that these six were novel O-AGCs.

A BLAST search of the NCBI database revealed that the OgN10 O-AGC is similar to that of *S. enterica* O42, and the OgN9 O-AGC was almost identical to that of *S. boydii* type 13 (**Figure 3C**). OgN10 and OgN9 strains agglutinated with *S. enterica* O42 and *S. boydii* type 13 antisera, respectively (data not shown).

### Primers for Identifying Novel O Genotypes

**FIGURE 4:**
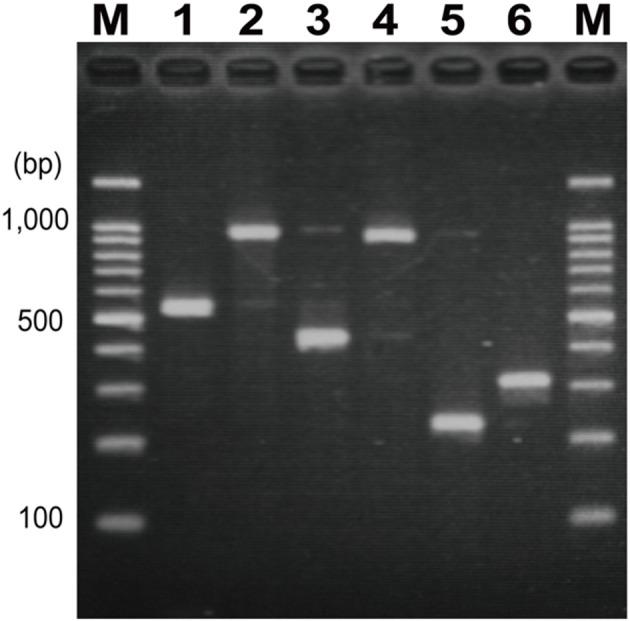
**Polymerase chain reaction (PCR) products of OgN strains by each specific-primer pair.** (1) OgN1 (525 bp), (2) OgN8 (940 bp), (3) OgN9 (423 bp), (4) OgN10 (892 bp), (5) OgN12 (232 bp), (6) OgN31 (311 bp), M; 100-bp DNA ladder markers.

Six PCR primer pairs were designed for identifying the novel O-genotypes (**Table 2** and **Figure 4**). All primer pairs were targeted unique sequences of *wzy*, except for OgN31 for which primers were targeted to a glycosyltransferase gene. Each PCR was evaluated by using all 185 O-serogroup reference strains from O1 to O188 and six novel O-genotype strains (listed in **Table 1**). PCR products of the expected sizes on the agarose gel were obtained only with the corresponding strains, and no extra products were observed in the size range between 100 and 1,500 bp (data not shown).

### Distribution of Novel O-Genotypes

Among 35 O-serogrouped *E. coli* strains whose O genotypes were not identified by the ECOG-PCR, five O141, three O1, three O39, one O40, and one O140 strains were classified by using the novel O-genotype PCR into OgN8, OgN10, OgN31, OgN1, and OgN12, respectively (**Table 3**). All 13 strains classified into five novel O-genotypes were *eae*-negative STEC, and OgN8, OgN10, and OgN31 had been isolated from patients with bloody diarrhea. OgN8 and OgN12 strains also cross-reacted with O41 and O34 antisera, respectively.

**Table 3:** List of STEC strains classified into novel O genotypes by PCR screening.

Strain ID	Source (case*^a^*)	Year	O serogroup	O-genotype	Novel O genotype*^b^*	*stx1*	*stx2*	*eae*
121862	Human (D)	2012	O40	OgUT	OgN1^∗^	-	+	-
102755	Human (BD)	2010	O141	OgUT	OgN8^∗^	-	+	-
110906	Human (AC)	2011	O141	OgUT	OgN8	-	+	-
133345	Human (AC)	2013	O141	OgUT	OgN8	+	+	-
PV10-60	Food	2010	O141	OgUT	OgN8	-	+	-
PV12-70	Food	2012	O141	OgUT	OgN8	-	+	-
072583	Human (BD)	2007	O1	OgUT	OgN10	-	+	-
090823	Human (D)	2009	O1	OgUT	OgN10^∗^	+	-	-
091971	Human (AC)	2009	O1	OgUT	OgN10	+	-	-
091275	Human (AC)	2009	O140	OgUT	OgN12	+	-	-
100998	Human (BD)	2010	O39	OgUT	OgN31^∗^	-	+	-
100999	Human (D)	2010	O39	OgUT	OgN31	-	+	-
NBK#585	Human (AC)	–	O39	OgUT	OgN31	-	+	-

*^a^AC, asymptomatic carrier; D, diarrhea; BD, bloody diarrhea. ^b^Strains used for sequencing of the O-AGC are indicated by asterisks. All strains were isolated in Japan.*

## Discussion

In this study, six novel O genotypes were revealed from STEC strains isolated from human patients, and the prevalence of these O genotype strains was confirmed in STECs. The OgN10 strains were serologically classified into the O1 serogroup. The O1-serogroup strain is often seen in extra-intestinal pathogenic *E. coli* from patients with urinary tract infections (Abe et al., 2008; Mora et al., 2009) and septicemic disease (Mora et al., 2009), and in avian pathogenic *E. coli* (Mora et al., 2009; Johnson et al., 2012). Our previous study showed that an O1 strain isolated from patient blood was classified into Og1 (Iguchi et al., 2015b) and sequence comparisons showed that both an APEC O1 strain from avian colibacillosis (Johnson et al., 2007) and the G1632 strain from a patient with a urinary tract infection (Li et al., 2010) carried the Og1-type O-AGC, whereas three STEC O1 strains used in this study were all classified into OgN10. Actually, we confirmed that five STEC O1 strains from cattle used in a previous study, described as O1B type (Mekata et al., 2014) were also classified into OgN10 (data not shown). In fact, *E. coli* O1 strains could be generally subtyped into two genotypes, Og1 and OgN10, which were clearly linked to extra-intestinal/avian pathogenic *E. coli* and STEC, respectively. Among the O1 serogroup, three types of antigen structures have so far been reported (Baumann et al., 1991; Gupta et al., 1992) and the β-linked side-chain *N*-acetyl-D-mannosamine residue was suggested to be a common O1-specific epitope (Gupta et al., 1992). A partial kinship of O antigen structure synthesized from different O-AGCs may be serologically recognized as the same O serogroup and may also be represented in OgN1 OgN8, OgN12, and OgN31 strains, related to O40, O141, O140, and O39, respectively. The subdivision within each O-serogroup based on the O-AGC DNA sequences may be useful for obtaining more reliable information for epidemiological studies of pathogenic *E. coli*. Another advantage for DNA-based typing is that serologically untypeable and ambiguous strains could be clearly classified. At the present time, the PCR-based method reported here is the only way to distinguish OgN9. Although, STEC OgN groups have not emerged as a major public health issue, these groups are believed to be a possible cause of diarrhea and bloody diarrhea. To gain more information about trends in STEC OgNs epidemiology, further studies of global OgN isolates are needed. The PCR method described in this study may help the surveillance and monitoring of the OgN groups. Additionally, published sequences from OgN O-AGCs may be used for other DNA-based methodologies, such as *in silico* typing using whole genome sequencing data (Joensen et al., 2015).

## Author Contributions

Conceived and designed the experiments: AI, MO, and TH. Performed the experiments: AI, SI, KS, and HN. Analyzed the data: AI and YO. Contributed reagents/materials/analysis tools: SI, KS, MO, and HM. Wrote the paper: AI. Critical revision of the paper for important intellectual content: AI, SI, and TH.

## Conflict of Interest Statement

The authors declare that the research was conducted in the absence of any commercial or financial relationships that could be construed as a potential conflict of interest. The reviewer BQ and handling Editor declared their shared affiliation and the handling Editor states that the process nevertheless met the standards of a fair and objective review.

## Footnotes

1 http://www.ssi.dk/English.aspx
